# Putative New West Nile Virus Lineage in *Uranotaenia unguiculata* Mosquitoes, Austria, 2013

**DOI:** 10.3201/eid2012.140921

**Published:** 2014-12

**Authors:** Karin Pachler, Karin Lebl, Dominik Berer, Ivo Rudolf, Zdenek Hubalek, Norbert Nowotny

**Affiliations:** University of Veterinary Medicine Vienna, Vienna, Austria (K. Pachler, K. Lebl, D. Berer, N. Nowotny);; Academy of Sciences, Brno, Czech Republic (I. Rudolf, Z. Hubalek);; Sultan Qaboos University, Muscat, Oman (N. Nowotny)

**Keywords:** West Nile virus, WNV, lineage 9, lineage 4, Uranotaenia unguiculata, flavivirus, phylogenetic analysis, Austria, mosquitoes, viruses

## Abstract

West Nile virus (WNV) is becoming more widespread and markedly effecting public health. We sequenced the complete polyprotein gene of a divergent WNV strain newly detected in a pool of *Uranotaenia unguiculata* mosquitoes in Austria. Phylogenetic analyses suggest that the new strain constitutes a ninth WNV lineage or a sublineage of WNV lineage 4.

West Nile virus (WNV), the most widespread flavivirus, is distributed throughout Africa, Asia, Europe, and Australia, and since 1999, WNV has also been present in the Americas (*1*). Within the last 2 decades, WNV infection has caused an increasing number of cases of neuroinvasive disease in humans and become a substantial public health problem (*1*).

Up to 8 lineages of WNV, based on genetic differences, have been proposed (*1*,*2*) (Table 1). Lineage 1 is widely distributed and further divided into lineage 1a, which includes the American strains; lineage 1b, which is also referred to as Kunjin virus and mainly described in Australia; and lineage 1c, which is also referred to as lineage 5 and comprises isolates from India. Lineage 2 has been detected in Africa and several parts of Europe, lineage 3 (Rabensburg virus) has been isolated only in the Czech Republic, and lineage 4 has been reported from Russia (*3*). A putative sixth lineage, based on a small genome fragment, has been described from Spain (*4*), and putative lineages 7 (Koutango virus) and 8 have been reported from Senegal (*2*).

**Table 1:** Overview of West Nile virus lineages

Lineage	Representative strain, location	GenBank accession no.
1a	NY 2000-crow3356, New York, USA	AF404756
1b	Kunjin virus, Australia	D00246
1c/5	804994, India	DQ256376
2	Goshawk-Hungary/04, Hungary	AAZ91684
3	Rabensburg virus 97–103, Czech Republic	AY765264
4/4a	LEIV-Krnd88–190, Russia	AY277251
6/4b, putative*	HU2925/06, Spain	GU047875
7 (Koutango virus), putative	Dak-Ar-D-5443, Senegal	EU082200
8, putative*	ArD94343, Senegal	KJ131502
9/4c, putative	WNV-Uu-LN-AT-2013, Austria	KJ831223

*Only partial sequence available.

WNV is maintained in an enzootic cycle between mosquitoes and wild birds (*1*). In 2013, ≈100 *Uranotaenia unguiculata* Edwards, 1913, mosquitoes were trapped during mosquito-monitoring projects at Lake Neusiedl-Seewinkel National Park in Austria and near Sedlec in the Czech Republic. In Russia, *Ur. unguiculata* mosquitoes have been described as hosting lineage 4 WNV strains (A. Platonov, unpub. data) (GenBank accession nos. FJ154906–49 and FJ159129–31). To determine whether *Ur. unguiculata* mosquitoes in Austria and the Czech Republic also host WNV, we investigated the mosquitoes collected in 2013 for the presence of WNV, focusing on lineage 4 viruses.

## The Study

During May–October 2013, ≈11,300 female mosquitoes belonging to 13 species were trapped at 4 sites in Lake Neusiedl-Seewinkel National Park in Burgenland State, Austria. Mosquito species were determined according to morphologic criteria (*5*). Individual mosquitoes were pooled by species and collection site and date. A total of 47 *Ur. unguiculata* mosquitoes were collected in Austria (12 pools, 1–12 mosquitoes/pool). The relative abundance of *Ur. unguiculata* mosquitoes among the total collected in Austria was 0.42%. During August 2013, ≈39,000 mosquitoes were trapped at 2 fish ponds (Nesyt and Novy) in Sedlec, Czech Republic, near the border with northeastern Austria. A total of 47 female *Ur. unguiculata* mosquitoes were grouped into 4 pools (2 with 1 mosquito each, 1 with 4 mosquitoes, and 1 with 41 mosquitoes). The relative abundance of *Ur. unguiculata* mosquitoes among the total collected in the Czech Republic was 0.12%.

The mosquito pools were homogenized in RNase-free water, and RNA was extracted by using the QIAamp Viral RNA Mini Kit (QIAGEN, Valencia, CA, USA). The samples were screened for the presence of flavivirus nucleic acid by reverse transcription PCR, using universal flavivirus primers MAMD (*6*) and CFD2 (*6*,*7*) for amplification of a partial nonstructural protein (NS) 5 sequence. Results were negative for the samples from Czech Republic. One pooled sample from Austria was positive; the pool contained 9 mosquitoes that had been captured in late August in Illmitz, a village east of Lake Neusiedl (47.769997°N, 16.752887°E). We obtained the complete polyprotein coding sequence and partial 5′ and 3′ noncoding ends of this novel WNV strain (GenBank accession no. KJ831223), which was designated West Nile virus-*Uranotaenia unguiculata*-Lake Neusiedl-Austria-2013 (WNV-Uu-LN-AT-2013). Primer sequences and amplification protocols are available upon request.

The complete polyprotein gene sequence of WNV-Uu-LN-AT-2013 shares a maximum identity of ≈83% with lineage 4 WNV strains isolated from *Ur. unguiculata* mosquitoes and *Dermacentor marginatus* ticks in Russia (*3*). At the amino acid level, the entire polyproteins of WNV-Uu-LN-AT-2013 and the lineage 4 strains from Russia share ≈96% identity (Table 2). Compared with the Russian lineage 4 strains, a 1,813-nt fragment of the NS5-coding sequence for the putative lineage 6 WNV, isolated from *Culex pipiens* mosquitoes in Spain (*4*), shares slightly higher nucleotide and amino acid identities with WNV-Uu-LN-AT-2013 (Table 2).

**Table 2:** Sequence identities between the newly identified WNV strain from Austria, WNV-Uu-LN-AT-2013, and other strains representing different WNV lineages*

Strain/lineage†	Nucleotide identity or amino acid identity, %, by strain/lineage†‡										
	WNV-Uu-LN-AT-2013	1a	1b	1c/5	2	3	4	6 (Spain)§	7 (Koutango virus)	8¶	Usutu virus
WNV-Uu-LN-AT-2013		88.3	87.9	87.0	88.8	86.7	96.2	95.9	85.3	81.2	75.5
1a	76.2		97.6	93.4	94.0	90.4	88.6	91.7	89.2	92.4	76.3
1b	75.4	88.2		92.7	93.5	89.8	88.3	91.2	88.8	92.0	76.1
1c/5	76.3	80.5	79.7		92.1	88.8	87.4	89.1	87.7	91.2	76.1
2	77.0	79.8	79.6	79.1		90.9	89.2	92.6	89.3	92.0	76.0
3	75.9	78.3	77.3	77.3	78.7		87.0	91.4	86.6	89.2	75.5
4	82.8	76.6	76.0	76.2	76.9	76.5		95.0	85.5	81.0	74.7
6 (Spain)§	83.2	78.1	78.1	77.7	78.6	79.5	81.7		88.6	–	80.8
7 (Koutango virus)	75.1	77.7	77.4	77.0	77.8	76.3	75.6	78.0		86.8	75.3
8¶	72.7	78.4	78.0	77.3	78.4	77.7	72.6	–	77.4		76.3
Usutu virus	71.2	72.4	72.6	72.4	71.3	71.0	70.1	73.6	72.4	72.5	

*Alignments were performed by using ClustalW2 (http://www.ebi.ac.uk/Tools/msa/clustalw2/). WNV, West Nile virus; WNV-Uu-LN-AT-2013, West Nile virus strain *Uranotaenia unguiculata*-Lake Neusiedl-Austria-2013; –, comparison between lineages 6 and 8 was not possible because the available partial sequences do not cover the same nucleotide regions. †GenBank accession nos. are as follows for the polyprotein genes/polyproteins: WNV-Uu-LN-AT-2013 (KJ831223), lineage 1a (AF404756/AAM81752), lineage 1b (D00246/BAA00176), lineage 1c (DQ256376/ABC40712), lineage 2 (DQ116961/AAZ91684), lineage 3 (AY765264/AAW81711), lineage 4 (FJ159129/ACH99530), lineage 6 (Spain) (GU047875/ADD69956), lineage 7 (Koutango virus) (EU082200/ABW76844), lineage 8 (KJ131502/AHV83443), Usutu virus (AY453411/AAS59402). ‡Amino acid sequences (above the diagonal) and nucleotide sequences (below the diagonal) are based on complete polyprotein genes, with the exception of lineage 6 and 8 strains, for which only partial sequences were available. §Comparison was based only on partial NS5 gene sequences. ¶Comparison was based only on complete envelope protein gene sequences.

Phylogenetic neighbor-joining trees were generated with MEGA5 software, using ClustalW alignments, 1,000 replicates for bootstrap testing, and evolutionary distances computation with the p-distance model (*8*). One phylogenetic tree was constructed on the basis of the complete polyprotein-encoding nucleotide sequences of 32 WNV strains representing all previously described lineages for which complete polyprotein-encoding sequences are available. This tree also showed a close relationship between WNV-Uu-LN-AT-2013 and the lineage 4 WNV strains from Russia; however, the newly identified strain forms a distinct branch (Figure, panel A). A second phylogenetic analysis that included the proposed lineage 6 virus from Spain and that was based on 1,813-nt fragments of NS5 showed a close grouping of WNV-Uu-LN-AT-2013 virus from Austria, the virus from Spain, and the lineage 4 viruses from Russia; similarity was slightly higher between the viruses from Austria and Spain (Figure, panel B).

**Figure:**
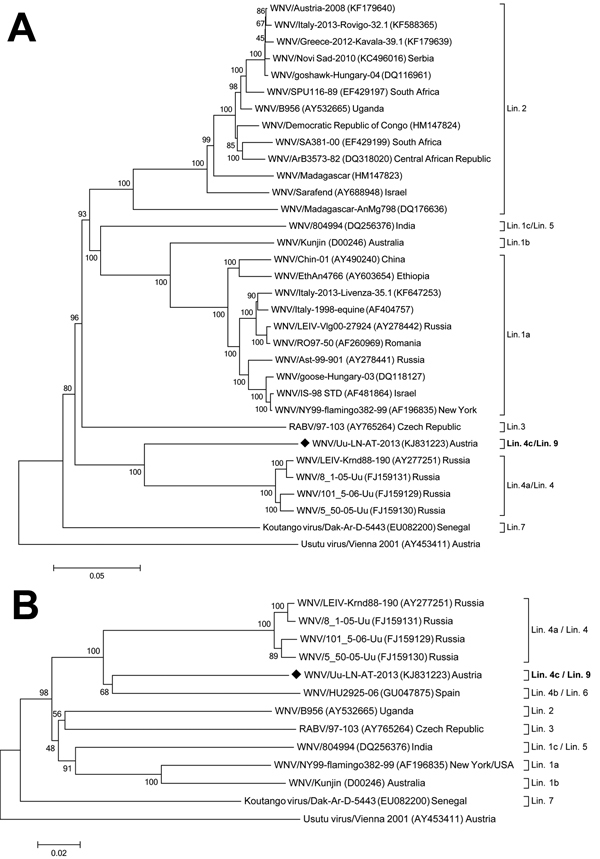
Phylogenetic positioning of WNV-Uu-LN-AT-2013, a West Nile virus (WNV) strain newly identified in Austria, within the species *West Nile virus*. A) Phylogenetic position as determined on the basis of the full-length polyprotein-coding nucleotide sequences. B) Phylogenetic position as determined on the basis of 1,813-nt fragments of NS5, which enabled inclusion of the proposed lineage 6 virus. The evolutionary history was inferred by using the neighbor-joining method of MEGA5 (*8*) with 1,000-fold bootstrap analysis, rooted against the respective sequence of Usutu flavivirus. Numbers next to the branches indicate the percentage of replicates in the bootstrap analysis. Black diamond indicates the WNV sequence determined in this study. GenBank accession numbers are shown in parentheses with the virus names. Scale bars indicate nucleotide substitutions per site. Lin., lineage; RABV, Rabensburg virus.

WNV-Uu-LN-AT-2013 encodes a polyprotein of 3,432 aa. The envelope protein carries 1 putative *N*-linked glycosylation site at asparagine residue N-154, which has been associated with increased WNV pathogenicity and neuroinvasiveness (*9*). The 3 highly conserved *N*-linked glycosylation sites at NS1 positions N-130, N-175, and N-207 in WNV strains were also calculated for WNV-Uu-LN-AT-2013 by using NetNGlyc 1.0 software (http://www.cbs.dtu.dk/services/NetNGlyc/). Glycosylation of NS1 at these 3 positions has been implicated in neuroinvasiveness (*10*), as has proline at NS1 aa position 250 (*11*), which is also present in WNV-Uu-LN-AT-2013. The NS2A-encoding nucleotide region contains a *foo* motif, which can mediate production of NS1′, a variant of NS1 that plays a role in neuroinvasiveness (*12*). A *fifo* motif, which has been described for the nonpathogenic mosquito-specific flaviviruses (*13*), could not be determined for WNV-Uu-LN-AT-2013.

## Conclusions

WNV lineages 1–4 and putative lineage 6 have been detected in Europe, but only WNV lineage 1a has spread across the American continents. Circulation of such a genetically diverse group of WNV strains in Europe may partly explain the epidemiologic differences observed between WNV disease in Europe and the Americas. In Europe, the presence of less pathogenic WNV strains may inhibit the spread of more pathogenic strains.

We propose that the WNV-Uu-LN-AT-2013 strain from Austria either constitutes a new lineage (lineage 9) or can be grouped into lineage 4 as sublineage 4c, with the strains from Russia and Spain as sublineages 4a and 4b, respectively. However, the short sequence available for the strain from Spain does not allow a clear-cut conclusion to be drawn with regard to lineage 4. We suggest that future designation of new WNV lineages should be restricted to viruses for which at least the complete polyprotein gene sequences have been determined. In addition, rules for defining virus lineages should be established by the International Committee on Taxonomy of Viruses.

Strain WNV-Uu-LN-AT-2013 has been detected only in *Ur. unguiculata* mosquitoes. These mosquitoes are mainly distributed in the southern half of Europe (*5*); in eastern Europe, they have spread from southern Ukraine and the Volga Delta through middle and southwestern Asia to Iran and Pakistan (*5*). In the Lake Neusiedl area of Austria, *Ur. unguiculata* mosquitoes seem to be an indigenous species, which was first reported in 1970 (*14*). In the Czech Republic, *Ur. unguiculata* mosquitoes have been detected only in Moravia, in the southern part of the country (*15*). Although there are anecdotal reports of *Ur. unguiculata* mosquitoes feeding on mammals, including humans, they feed mainly on amphibians and reptiles (*5*).

The pathogenicity of strain WNV-Uu-LN-AT-2013 in humans and animals has not been elucidated. Genetic data show that the strain carries typical WNV pathogenicity markers and suggest that WNV-Uu-LN-AT-2013 is not restricted to mosquitoes. Additional monitoring studies involving cell culture and animal isolation experiments are necessary to evaluate the pathogenic potential of this virus for humans and animals.
